# Rapid recurrence of primary gastric choriocarcinoma after complete resection

**DOI:** 10.1016/j.ijscr.2019.03.045

**Published:** 2019-03-30

**Authors:** Amane Hirotsu, Yoshihiro Hiramatsu, Sanshiro Kawata, Tomohiro Matsumoto, Yusuke Ozaki, Hirotoshi Kikuchi, Megumi Baba, Kinji Kamiya, Hiroyuki Konno, Hiroya Takeuchi

**Affiliations:** a Department of Surgery, Hamamatsu University School of Medicine, Hamamatsu, Japan; b Department of Perioperative Functioning Care and Support, Hamamatsu University School of Medicine, Hamamatsu, Japan; c Department of Emergency and Disaster, Hamamatsu University School of Medicine, Hamamatsu, Japan; d Hamamatsu University School of Medicine, Hamamatsu, Japan

**Keywords:** PGC, primary gastric choriocarcinoma, CT, computed tomography, β-hCG, β-human chorionic gonadotropin, Primary gastric choriocarcinoma, Total gastrectomy, Short-term survival

## Abstract

**Introduction:**

Primary gastric choriocarcinoma (PGC) is a rare and rapidly invasive tumor. We report a case of PGC diagnosed by endoscopic biopsy and treated with gastrectomy and chemotherapy.

**Presentation of case:**

A 78-year-old man was referred to our hospital because esophagogastroduodenoscopy showed a tumor at the fornix of the stomach. Pathologic examination of biopsy specimens revealed choriocarcinoma. Abdominal computed tomography (CT) revealed no enlarged abdominal lymph nodes or distant metastases. Robot-assisted total gastrectomy with spleen-preserving D2 lymphadenectomy was performed on the basis of a diagnosis of cT2N0M0, stage cIB PGC. The pathologic diagnosis was pT2, ly0, v1, pN0, PM0, DM0, stage pIB PGC. The postoperative course was uneventful, and the patient was followed carefully without adjuvant chemotherapy. Three months after gastrectomy, blood tests indicated that serum β-human chorionic gonadotropin (β-hCG) levels had increased, and CT revealed multiple liver metastases. The patient underwent a standard nongestational choriocarcinoma chemotherapy regimen with etoposide, methotrexate, actinomycin D, cyclophosphamide, and vincristine. Although β-hCG levels temporarily decreased with chemotherapy, the patient experienced tumor recurrence with ascites and his serological test demonstrated an elevated level of β-hCG (120 ng/mL). The patient died 10 months postoperatively.

**Conclusion:**

We report a case of stage pIB PGC with poor prognosis, recurring at only 3 months postoperatively despite curative surgery and chemotherapy.

## Highlights

• A 78-year-old man was diagnosed with stage cIB primary gastric choriocarcinoma.
• Total gastrectomy with spleen-preserving D2 lymphadenectomy was performed.
• Early recurrence was diagnosed 3 months postoperatively.
• The patient underwent a standard nongestational choriocarcinoma chemotherapy.

## 1 Introduction

Primary gastric choriocarcinoma (PGC) is a rare and rapidly invasive tumor [1], with a median survival of less than several months [2]. We report a case of stage pIB PGC causing recurrent liver metastasis as early as 3 months after curative surgery. This work has been reported in line with the SCARE criteria [3].

## 2 Presentation of case

**Fig. 1:**
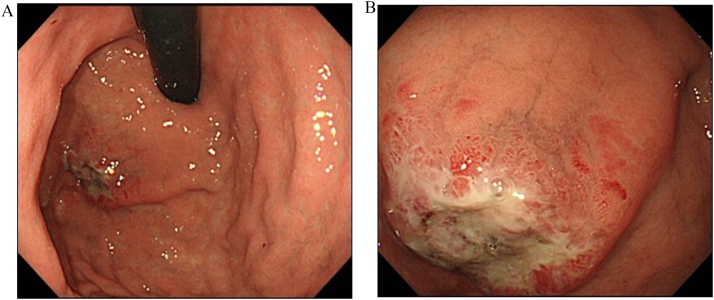
Esophagogastroduodenoscopy results. (a) A tumor was localized at the posterior wall of the fornix of the stomach. (b) The tumor was classified as a type 3 tumor (size: 25 mm).

**Fig. 2:**
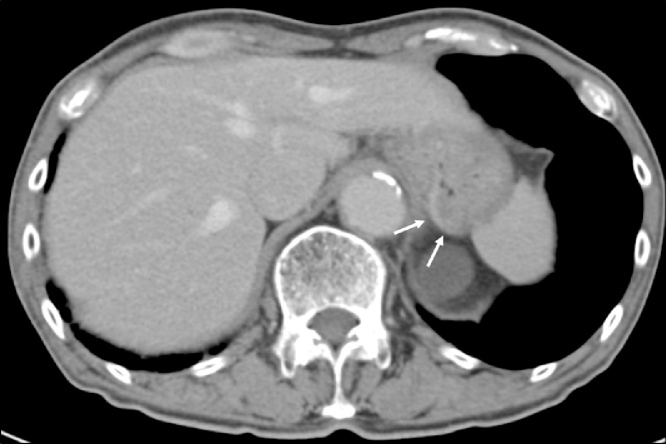
Abdominal computed tomography revealed thickening of the posterior wall of the upper part of the stomach with contrast effect (*arrow*) and no enlarged abdominal lymph nodes or distant metastases.

**Fig. 3:**
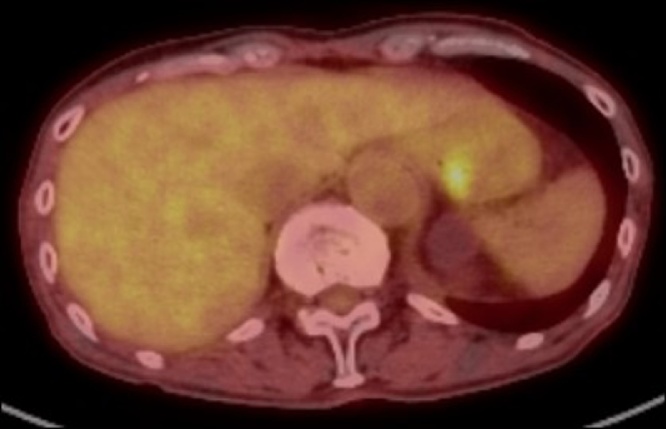
Preoperative positron emission tomography revealed accumulation of fluorodeoxyglucose at the tumor site (standardized uptake value, max 5.4) and no significant accumulation at any other sites.

**Fig. 4:**
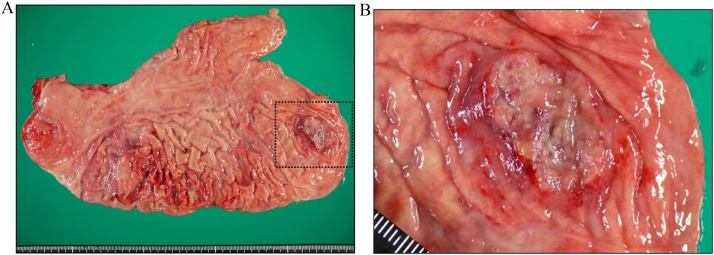
(a) Robot-assisted total gastrectomy with spleen-preserving D2 lymphadenectomy was performed. (b) A type 3 tumor with an unclear border resected from the fornix of the stomach.

**Fig. 5:**
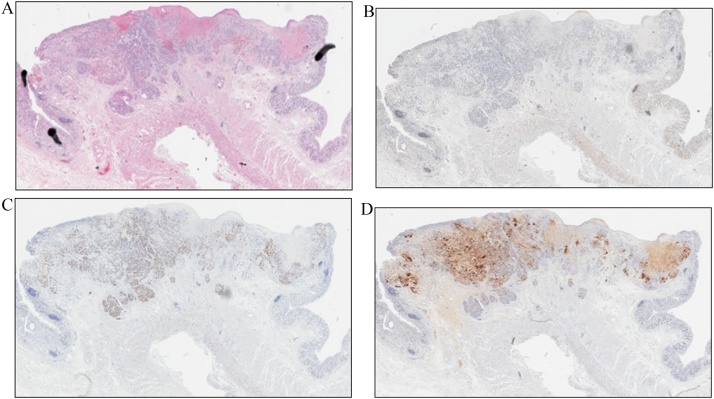
Histologic findings of resected specimens: (a) Hematoxylin and eosin ×10, (b) p40 × 10, (c) Sal-like protein 4 × 10, and (d) human chorionic gonadotropin ×10.

A 78-year-old man was referred to our hospital because esophagogastroduodenoscopy showed a tumor at the fornix of the stomach. A biopsy at our institution confirmed a type 3 tumor 25 mm in size approximately 3 cm from the esophagogastric junction (Fig. 1). The pathologic diagnosis of the biopsy specimen was choriocarcinoma. The tumor was positive for p40, Sal-like protein 4 (SALL4), and human chorionic gonadotropin (hCG). SALL4 is a marker of germ cell tumors, which is the common presentation of extragonadal PGC. Moreover, PGC consists of tumor cells similar to trophoblast cells of the placenta villi, which are positive for β-hCG. Therefore, these immunohistochemical findings confirmed the diagnosis of PGC. Levels of the tumor marker serum β-hCG were mildly elevated at 3.9 ng/mL. Abdominal computed tomography (CT) showed thickening of the stomach wall with contrast effect on the posterior side of the upper part of the stomach. There were no clear findings of direct invasion to the surrounding area, lymphatic metastasis, or distant metastasis (Fig. 2). The tumor was diagnosed as cT2N0M0, stage cIB PGC, and was considered resectable. Given that PGC is known for its postoperative high recurrence rate and poor prognosis, we performed positron-emission tomography (PET), which showed accumulation of fluorodeoxyglucose in the tumor site [standardized uptake value max 5.4]. Otherwise, there was no remarkable accumulation at other sites (Fig. 3). In addition, a second CT performed 1 month later showed no increase in the size of the primary tumor or appearance of new lesions, and no increase in serum β-hCG was detected. Therefore, robot-assisted total gastrectomy with spleen-preserving D2 lymphadenectomy was performed (Fig. 4). The operative time was 747 min, and the estimated intraoperative blood loss was 20 mL. The resected specimen was weakly positive for p40, strongly positive for SALL4, and strongly positive for β-hCG (Fig. 5). The diagnosis was choriocarcinoma with some tubular components in the mucous membranes. The pathologic diagnosis was pT2, ly0, v1, pN0, PM0, DM0, stage pIB PGC. The patient was discharged 13 days after the surgery with no complications.

Because the patient had stage pIB disease and because the effectiveness of tegafur/gimeracil/oteracil (TS-1) for choriocarcinoma is unknown, postoperative adjuvant chemotherapy was not performed. Since serum β-hCG has been reported to be a useful marker for postoperative recurrence, he was followed carefully with monthly serum β-hCG measurements. Serum β-hCG gradually began to increase 2 months postoperatively and reached 120 ng/mL 3 months postoperatively. At the same time, PET revealed multiple liver metastases, and early recurrence was diagnosed. The patient received the standard nongestational choriocarcinoma chemotherapy regimen of etoposide, methotrexate, actinomycin D, cyclophosphamide, and vincristine (EMA/CO). Although β-hCG decreased temporarily, it gradually increased again. The liver metastases and ascites also increased. The patient died 10 months after the resection of the primary tumor.

## 3 Discussion

Choriocarcinoma can be gonadal or extragonadal in origin and occurs most frequently in the uterus in association with pregnancy. The most common sites for extragonadal tumors are the mediastinum, ovary, and testis [4]. PGC is an hCG-producing epithelial tumor with differentiation [1] first described by Davidsohn in 1905, and approximately 140 cases have been reported in the international medical literature to date [5]. However, it accounts for only approximately 0.08% of all gastric cancers [6]. Pure choriocarcinoma is especially rare, and mixed types that consist of choriocarcinoma and adenocarcinoma are more frequent [7,8]. Some studies report that PGC is accompanied by adenocarcinoma and exhibits a gradual transition from adenocarcinoma component to choriocarcinoma component [9]. Several studies revealed that the pathogenesis of PGC can be explained by dedifferentiation of malignant adenocarcinoma tissue to the level of the ectoderm, retaining the ability to form trophoblasts [10]. The dedifferentiation theory was proposed by Pick in 1926 and is widely accepted, although the pathogenesis of PGC remains controversial [11,12]. The clinical features of PGC are similar to those of gastric adenocarcinoma: the mean age, male-to-female ratio, and tumor location of PGC all parallel those of gastric adenocarcinoma [13]. Due to the coexistence of adenocarcinoma and choriocarcinoma components, PGC is often misdiagnosed as adenocarcinoma if diagnosed only by hematoxylin and eosin staining [13]. Appropriate use of β-hCG immunostaining is required to make the correct diagnosis [14].

PGC has a poor prognosis, high mortality rate, and short overall survival rate, unlike primary gastric adenocarcinoma. However, PGC has an overall 5-year survival rate of 50% for patients who present earlier with resectable disease [15]. Kobayashi et al. [9] reported that synchronous liver metastasis, residual postoperative tumor, and absence of chemotherapy were significant prognostic parameters of short overall survival. They also suggested that PGC with a high metastatic potential, especially to the liver, may have the highest malignant potential among metastatic PGCs [9]. However, one study also reported long-term survival obtained by excising a single liver metastasis [16].

PGC is a highly invasive and widely metastasizing tumor [1]. The tumor growth rate is much faster than that of gastric adenocarcinoma, and the doubling time is reported to be approximately 3 weeks [17]. Approximately 30% of patients already have metastatic disease at diagnosis [18]. The lymph nodes are the most common site of metastasis (87%), followed by the liver (45%), peritoneum (23%), and lung (8%) [4].

Surgical indications for PGC should be considered carefully. If bleeding from the tumor is observed, palliative surgery is considered, followed by chemotherapy, but no effective standard treatment regimen for PGC has been established [19]. In one case, the standard nongestational choriocarcinoma chemotherapy regimen EMA/CO resulted in a complete serological response after four cycles [14]. In our case, treatment with EMA/CO was started at the time of recurrence, but a sufficient effect was not obtained. We next planned to administer TS-1, which is the usual treatment for gastric cancer, but we could not do so because of the patient's worsening performance status. However, even if TS-1 could have been administered, we assume that the effect would have been minimal, as only the adenocarcinoma component, not the choriocarcinoma component, would have been likely to respond. Tissue biopsy of a recurrent metastatic lesion can diagnose the histologic type of the metastatic lesion and is expected to help with regimen selection, but PGC is highly invasive. Moreover, serum β-hCG has been reported to serve as a postoperative serum marker of tumor recurrence [14]. In our case, serum β-hCG was measured every month and was useful as a marker reflecting the postoperative recurrence. However, more studies and larger clinical trials are needed to define both an appropriate standard treatment and determine better mechanisms for patient follow-up.

## 4 Conclusion

We reported a case of stage pIB PGC with poor prognosis, recurring at only 3 months postoperatively despite curative surgery and chemotherapy.

## Conflicts of interest

The authors declare no conflict of interest.

## Sources of funding

This research did not receive any specific grant from funding agencies in the　public, commercial, or not-for-profit sectors.

## Ethical approval

Ethical approval was not required and patient identifying knowledge was not presented in the report.

## Consent

Written informed consent was obtained from the patient for publication of this case report and accompanying images. Patient’s names, initials, or hospital numbers is not used. Identified information in the images of the patient is not also used and he has a right to privacy.

## Author contribution

Yoshihiro Hiramatsu performed the surgical intervention. Amane Hirotsu participated to the surgical intervention. Amane Hirotsu, Yoshihiro Hiramatsu, Sanshiro Kawata, Tomohiro Matsumoto, Yusuke Ozaki, Hirotoshi Kikuchi, Kinji Kamiya, and Megumi Baba contributed in the collection of the data. Amane Hirotsu drafted and edited the manuscript. Hiroyuki Konno and Hiroya Takeuchi contributed in study concept and design.

## Registration of research studies

Clinical evaluation of the safety of robotic gastrectomy using da Vinci surgical system (DVSS) for resectable gastric cancer (UMIN000019366).

## Guarantor

Yoshihiro Hiramatsu.

## Provenance and peer review

Not commissioned, externally peer-reviewed.
